# *In Vitro* and *In Vivo* Trypanocidal Activity of H_2_bdtc-Loaded Solid Lipid Nanoparticles

**DOI:** 10.1371/journal.pntd.0002847

**Published:** 2014-05-08

**Authors:** Zumira A. Carneiro, Pedro I. da S. Maia, Renata Sesti-Costa, Carla D. Lopes, Tatiana A. Pereira, Cristiane M. Milanezi, Marcelo A. Pereira. da Silva, Renata F. V. Lopez, João S. Silva, Victor M. Deflon

**Affiliations:** 1 Faculdade de Ciências Farmacêuticas de Ribeirão Preto, University of São Paulo, Ribeirão Preto, São Paulo, Brazil; 2 Instituto de Química de São Carlos, University of São Paulo, São Carlos, São Paulo, Brazil; 3 Departamento de Bioquímica e Imunologia, School of Medicine, University of São Paulo, Ribeirão Preto, São Paulo, Brazil; 4 Instituto de Física de São Carlos, University of São Paulo, São Carlos, São Paulo, Brazil; 5 Centro Universitário Central Paulista - UNICEP, São Carlos, São Paulo, Brazil; Northeastern University, United States of America

## Abstract

The parasite *Trypanosoma cruzi* causes Chagas disease, which remains a serious public health concern and continues to victimize thousands of people, primarily in the poorest regions of Latin America. In the search for new therapeutic drugs against *T. cruzi*, here we have evaluated both the *in vitro* and the *in vivo* activity of 5-hydroxy-3-methyl-5-phenyl-pyrazoline-1-(S-benzyl dithiocarbazate) (H_2_bdtc) as a free compound or encapsulated into solid lipid nanoparticles (SLN); we compared the results with those achieved by using the currently employed drug, benznidazole. H_2_bdtc encapsulated into solid lipid nanoparticles (a) effectively reduced parasitemia in mice at concentrations 100 times lower than that normally employed for benznidazole (clinically applied at a concentration of 400 µmol kg^−1^ day^−1^); (b) diminished inflammation and lesions of the liver and heart; and (c) resulted in 100% survival of mice infected with *T. cruzi*. Therefore, H_2_bdtc is a potent trypanocidal agent.

## Author Summary

The protozoan parasite *Trypanosoma cruzi* causes Chagas disease, a condition that affects the poorest regions of Latin America mainly. The chronic phase of this disease disables thousands of patients, constituting an important public health issue. The pharmacotherapy that is currently applied to treat the disease emerged many decades ago, is ineffective in most patients, mainly during the chronic phase, and has serious side effects. In a recent study, we showed that the compound 5-hydroxy-3-methyl-5-phenyl-pyrazoline-1-(S-benzyldithiocarbazate) (H_2_bdtc) is a potential drug candidate against the *in vitro* trypomastigote form of Tulahuen strains of *T. cruzi*. Here we report that H_2_bdtc loaded into solid lipid nanoparticles (H_2_bdtc-SLNs) displays good trypanocidal activity against the trypomastigote form of the Y strain of *T. cruzi* both *in vitro* and *in vivo*. Our *in vivo* experiments revealed that H_2_bdtc-SLN is 100 times more active than benznidazole (BZN), the drug that is commercially available to treat Chagas disease. Surprisingly, this compound has no side effects on the *T. cruzi* acute phase. Hence, we propose that H_2_bdtc-SLNs possesses interesting anti-Trypanosoma properties.

## Introduction

*T. cruzi* parasites are transmitted by insect vectors (triatomine bugs). T.cruzi is the causative agent of Chagas disease, which is silent and can remain asymptomatic for years [1], [2], [3]. A century after its discovery, this disease remains a serious public health issue—it is closely associated with human poverty and political instability as well as with little investment in drug development. According to the World Health Organization (WHO), between seven and eight million people are infected with *T. cruzi* worldwide, primarily in Latin America [4], [5], [6]. One in every four Chagas patients develops a fatal symptom of the disease due to lack of adequate diagnosis and treatment.

Nifurtimox and benznidazole (BZN) are currently available to treat the disease [7], [8], [9], [10]. However, neurological side effects have led commercial nifurtimox production to be discontinued [11]. As for BZN, although it is mainly effective during the acute phase of the infection, it presents undesirable side effects such as rash and gastrointestinal symptoms [12], so patients often fail to comply with the treatment [8]. Long treatment periods (30, 60, or 90 days) and appropriate pediatric formulations not available (administration of the medication to children often requires tablet fractionation) also limit BZN use [9], [11], [13]. A further concern is that no effective treatment for the symptomatic chronic phase of Chagas disease exists, so the patients usually receive palliative drugs at this stage [14], [15]. Therefore, a number of researchers are making considerable efforts to find new drugs to combat this disease.

**Figure 1:**
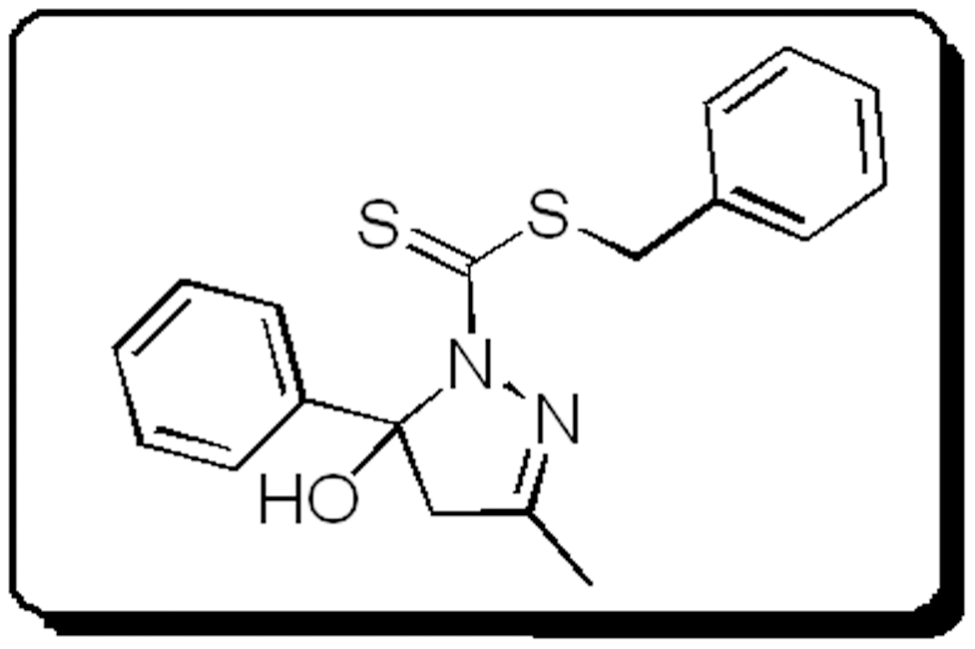
General molecular structure of the new cyclic S-dithiocarbazate derivatives.

Dithiocarbazates display notable biological and pharmacological properties, including anticancer [16], [17], antimicrobial [17], [18], [19], and insecticidal activities [20]. A recent study has shown that cyclic compounds derived from S-dithiocarbazate and 1,3-diketones exhibit significant trypanocidal activity [21]: in particular, 5-hydroxy-3-methyl-5-phenyl-pyrazoline-1-(S-benzyldithiocarbazate) (previously referred to as H_2_L^2a^ [21]) which was renamed H_2_bdtc in this reference in this work (Figure 1) constitutes a potential drug lead to develop a new agent against the trypomastigote form of Tulahuen strains of *T. cruzi* [21]. Nevertheless, the lipophilic character of H_2_bdtc may limit its administration and result in low oral bioavailability [22].

Drug delivery systems can help to circumvent this problem. Because lipids have excellent physiological acceptability and can promote drug absorption as well as selective lymphatic uptake, researchers have focused on lipid-based drug release systems [23]. In particular, solid lipids constitute solid lipid nanoparticles (SLNs) at room and body temperature. Since SLNs consist of biocompatible and biodegradable lipids with low or no human toxicity, they can function as drug delivery systems [24], [25]. SLNs offer many advantages: they protect the drug against degradation, enable controlled drug release, and dismiss the use of organic solvents. Moreover, SLNs can be produced on a large scale, meeting industrial requirements [24], [26].

Our group has used H_2_bdtc *in vitro* experiments involving the Tulahuen strain (group I) [21]. The resistances of this group and of the Y strain (group II) have been reported to be different, based on phosphatase activities in T. cruzi homogenates [27]. Tulahuen had an optimum phosphatase activity at pH 4.0 and the Y strain at pH 7.0 [27]. Also in chronic phase has been associated with *T*. cruzi II-restricted infections [28]. In this sense, evaluating the trypanocidal activity of H_2_bdtc against the Y strain could support the use of this compound as a new drug against *T. cruzi*. In addition, so far little attention has been paid to the use of SLNs to treat Chagas disease [29], [30]. Therefore, this work investigates the *in vitro* and *in vivo* trypanocidal activity of free H_2_bdtc and H_2_bdtc encapsulated into SLNs (H_2_bdtc-SLNs) against the Y strain of *T. cruzi* and compares results with data obtained for the currently available drug BZN.

## Materials and Methods

### General

BZN, a product manufactured by Lafepe, Brazil, was used as a reference drug. The synthesis of H_2_bdtc has been described previously [21]. RPMI Medium 1640 supplemented with 5% bovine fetal serum (GIBCO, Grand Island, NY, USA), 100 IU mL^−1^ penicillin G, and 100 mg mL^−1^ streptomycin (Gibco-BRL, Grand Island, NY, USA) was employed. Dimethyl sulfoxide (DMSO) and propidium iodide (PI) were obtained from Sigma-Aldrich Chemicals Co. (St. Louis, MO, USA). Stearic acid and sodium taurodeoxycholate were purchased from Sigma-Aldrich (St. Louis, MO, USA), Lipoid S 100 (soya lecithin) was acquired from Lipoid (Ludwigshafen, KOLN, Germany), and Amicon Ultra 15, MWCO 100 K, was provided by Millipore (Billerica, MA, USA).

### Preparation of H_2_bdtc-loaded solid lipid nanoparticles (H_2_bdtc-SLNs)

SLNs were prepared using a microemulsion method [31]. Briefly, the desired amount of sodium taurodeoxycholate (0.12% w/v) was dissolved in hot aqueous phase, which was added to melted stearic acid (0.95% w/v) containing soya lecithin (0.48% w/v) and H_2_bdtc (0.02% w/v). The mixture was emulsified via magnetic stirring at 90.0±2.0°C, until a thermodynamically stable microemulsion formed. The SLNs dispersion was obtained by cooling the hot microemulsion in cold water (2–5°C) under vigorous stirring at 20,000 rpm for 10 min (IKA-T25 Ultra-turrax, Germany) at 1∶20 ratio (microemulsion/cold water). Next, the SLNs aqueous dispersion was subjected to high-pressure homogenization (EmulsiFlex – C3, Germany) at 500 bars for 10 min.

### Particle size, dispersity index (D-stroke), and zeta potential determination

The particle size and dispersity of the H_2_bdtc-SLNs dispersion were measured via photon correlation spectroscopy (PCS) [32]; the zeta potential was determined on the basis of the electrophoresis mobility of the nanoparticles using the Zetasizer ZS Nano 90 (Malvern Instruments, UK.). The samples were diluted (1∶10) with distilled water at 25.0°C.

### Atomic Force Microscopy (AFM)

The morphology of H_2_bdtc-SLNs was assessed using an atomic force microscope (ICON Bruker, USA). The samples were prepared by immersing freshly cleaved mica (Muscovite Mica Substrates Sheets, SPI Supplies, China) in SLNs aqueous dispersion and stored overnight, at room temperature, to complete the drying process. The samples were evaluated by AFM in the intermittent contact mode (tapping mode) by scanning the surface of mica (2 µm×2 µm in area) using a rectangular silicon cantilever with a spring constant of 40 N m^−1^ vibrating at a frequency of 320 kHz. Imaging was performed at room temperature, and the topology image was used to determine the morphology of H_2_bdtc-SLNs [33].

### Drug entrapment efficiency (EE%) determination

The total H_2_bdtc content in the H_2_bdtc-SLNs was determined by UV-vis spectroscopy at 400 nm (UV Spectrophotometer UV 1800, Shimadzu, Japan). First, a defined amount of H_2_bdtc-SLNs was dissolved in dimethyl sulfoxide. The amount of encapsulated drug was indirectly measured after centrifuging the H_2_bdtc-loaded SLNs for 40 min at 6000 rpm (1605 G), at 25°C in a centrifuge (Heraeus Megafuge 16 R Thermo Scientific, USA) equipped with a membrane concentrator (Amicon Ultra 15, MWCO 100 K, Millipore Corporation, USA). The filtrate was diluted with dimethyl sulfoxide (1∶1), and the concentration of free H_2_bdtc in the diluted filtrate was determined using the same conditions employed to measure the total H_2_bdtc content used during the loading procedure (section 2.2). The amount of H_2_bdtc loaded into SLNs was calculated by subtracting the amount of free H_2_bdtc in the filtrate from the total amount of H_2_bdtc used during loading (26). EE (%) was determined using the following equation [26], [34]. 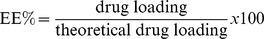



### Partition coefficient of H_2_bdtc (K _octanol/water_)

Partition coefficients for the H_2_bdtc were determined in triplicate in an n- octanol/water system following a published procedure [35]. Measurements of H_2_bdtc n-octanol/water partition coefficients were carried out using the shake-flask method. H_2_bdtc was dissolved in aqueous solution previously saturated with n-octanol at a concentration of 1 mg/mL and mixed with the same volume of octanol also previously saturated with water. Samples were stirred for 30 min, separate in two phases, and centrifugated for 10 min at 2000 rpm. The amount of H_2_bdtc in the aqueous phase was quantified by UV-visible spectroscopy.

### Mice

Female Swiss mice (6 to 8 weeks old) were bred and maintained at the Department of Biochemistry and Immunology, School of Medicine of Ribeirao Preto, University of São Paulo, Ribeirão Preto, Brazil. The mice were maintained in microisolator cages under standard conditions; they were fed with food and water ad libitum.

### Ethics statement

All the *in vivo* procedures were performed in accordance with the guidelines issued by the Brazilian College of Animal experimentation (COBEA) and received prior approval by the Ethics Committee on Animal Experimentation – CETEA (n° 006/2011) of the School of Medicine of Ribeirão Preto.”

### Parasites and experimental infection

All the experiments were conducted using the trypomastigote form of the Y strain of *T. cruzi* (Lineage type II). For the *in vitro* experiments, parasites were grown in a fibroblast cell line (LLC-MK2). For the *in vivo* experiments, mice were intraperitoneally inoculated with 2.0×10^3^ bloodstream trypomastigote forms, which had been derived from previously infected Swiss mice.

### *In vitro* evaluation of the trypanocidal activity and cytotoxicity of free H_2_bdtc and H_2_bdtc-loaded SLNs

The trypanocidal activities of free H_2_bdtc, H_2_bdtc-SLNs, and BZN against the trypomastigote form of the *T. cruzi* Y strains were evaluated as described previously [36]. To this end, the trypomastigote culture at a concentration of 6.5×10^6^ parasites mL^−1^ was re-suspended in RPMI 1640 medium with 5% FBS. Triplicate cultures were treated with one of the investigated drugs and maintained at 37.0±0.1°C in a humidified atmosphere of 5% CO_2_. To test parasite viability, the number of motile forms was determined using a previously described method [37]. The concentration of compound corresponding to 50% trypanocidal activity after 24 h of incubation was expressed as the IC_50try_ (inhibitory concentration for the trypomastigote form).

Spleen cells isolated from C57BL/6 mice, macerated in RPMI 1640 medium (Gibco), and filtered using a 100-µm pore filter were used to evaluate the cytotoxicity *in vitro*. The isolated cells were centrifuged at 1500 rpm for 10 min, and erythrocytes were lysed in lysis buffer for 5 min, at room temperature. Cells were washed, counted, and resuspended at 6.5×10^6^ mL^−1^ in RPMI medium containing 5% fetal bovine serum. The spleen cells were seeded to a 96-well microplate (n = 2) and incubated for 24 h with H_2_bdtc diluted in dimethyl sulfoxide (DMSO, final H_2_bdtc concentration not exceeding 0.5%) or H_2_bdtc-SLNs (concentrations ranging from 125 µM to 0.24 µM in serial dilutions). BZN (Roche) was used as the reference drug; Tween 20 was employed as positive control for cell death. After the incubation period, the cells were washed and incubated with propidium iodide at a final concentration of 10 µg mL^−1^. Cell cytotoxicity was measured on a flow cytometer (FACSCantoII - BD), and the data were analyzed using the FlowJo program (Tree Star).

### *In vivo* evaluation of the cytotoxicity and trypanocidal activity of free H_2_bdtc and H_2_bdtc-SLNs

Female Swiss mice aged between 6 and 8 weeks, weighing between 20 and 25 g, were infected with 2.0×10^3^ blood trypomastigotes per animal. A total of four experimental groups consisting of seven Swiss mice each were included in the study. Treatment started at day 5 post-inoculation (p.i.). BZN, free H_2_bdtc and H_2_bdtc-SLNs were orally administered at 4 µmol kg^−1^ (BZN 1.0 mg kg^−1^ day^-1^/free H_2_bdtc and H_2_bdtc-SLNs 1.4 mg kg^−1^ day^−1^) per day for 10 consecutive days. The following treatments were applied: Group 1 =  PBS control group; infected and not treated, Group 2 =  infected and treated with BZN, Group 3 =  infected and treated with free H_2_bdtc, and Group 4 =  infected and treated with H_2_bdtc-SLNs. To evaluate parasitaemia and mortality, seven animals from each group were used. Seven animals were killed at day 22 p.i. (early mortality), to quantify inflammation of the heart and liver and to measure creatine kinase-MB (CK-MB) and glutamic-pyruvic transaminase (GPT) production.

### Parasitemia and mortality

Parasitemia was analyzed on alternate days from day 7 p.i.; to this end, 5 µL of fresh blood was collected from the animal tail. The count of 100 fields was performed via direct observation under a light microscope [38]. Mortality was inspected on a daily basis until day 60.

### Histological analysis

Groups of seven mice were euthanized at day 20 p.i., and portions of the heart and liver were fixed in paraffin for histological analysis. To assess inflammatory infiltration via light microscopy DP71 (Olympus Optical Co, Japan), tissues were sectioned at a 5-µm thickness and stained with hematoxylin-eosin (H&E). Each tissue section was imaged 25 times, and the percentage of the area occupied by cellular infiltrates was determined using the Image J program.

### Quantitative real-time PCR (qPCR)

Quantitative PCR was used to determine the amount of parasitic DNA in heart tissues. Briefly, DNA was purified from 25 mg of heart tissue using a QIAamp DNA Mini Kit (Qiagen), according to the manufacturer's instructions. Each PCR reaction comprised 40 ng of genomic DNA; 0.3 µM of the *T. cruzi*-specific primers TCZ-F 5′-GCTCTTGCCCACAMGGGTGC-3′ (M = A or C) TCZ-R 5′-CCAAGCAGCGGATAGTTCAGG-3′ [39], which amplify a 182-bp product; 7.65 µL of GoTaq qPCR Master Mix; and H_2_O (final total volume of 15 µL).

The reactions were performed using the Real-Time PCR System. The cycling program involved a denaturation cycle of 95.0°C for 10 min, followed by 40 cycles of the three steps of the amplification phase: 95.0°C for 15 s, 55.0°C for 30 s, and 72.0°C for 15 s. The melting phase was performed at 95.0°C for 15 s and at 60.0°C for 1 min, followed by a 0.3°C ramp and then 95.0°C for 15 s. During the melting phase, the acquisition setting was set at step. The data were analyzed with StepOne Software version 2.2.2.

### Serum activity of creatine kinase isoform MB (CK-MB) and glutamic-pyruvic transaminase (GPT)

The cardiac and hepatic lesions of mice infected with *T. cruzi*, treated or not, were assessed by measuring the creatine kinase-MB (CK-MB) and glutamic-pyruvic transaminase (GPT) levels, respectively, in the serum at day 22 p.i. The CK-MB levels were measured using a CK-MB kit (Liquiform, Brazil), as previously described [40]. Absorbance was measured on a microplate spectrophotometer (EMAX Molecular Devices Corporation, California, EUA). The color produced from this reaction was measured at a wavelength of 340 nm; the results are expressed in U/I. GPT was analyzed using an ALT/GPT kit (Liquiform, Brazil), according to the manufacturer's instructions. The colorimetric assay determines the amount of pyruvate produced according to the Reitman and Frankel method, from the formation of 2,4-dinitrophenylhydrazine [41]. The color produced by this reaction was measured at a wavelength of 505 nm.

### Statistical analyses

Data are expressed as the mean SEM. Student's t-test was used to analyze the statistical significance of the variation between the infected and control assays. Differences were considered statistically significant when P<0.05. The differences in droplet size, dispersity, zeta potential, and entrapment efficiency values achieved during the stability test were evaluated via a one-way ANOVA analysis of variance followed by Tukey post-test analysis. The differences were considered statistically significant when P<0.05. All the analyses were performed using PRISM 5.0 software (Graph Pad, San Diego, CA, US).

## Results

### Preparation and characterization of H_2_bdtc-SLNs

**Figure 2:**
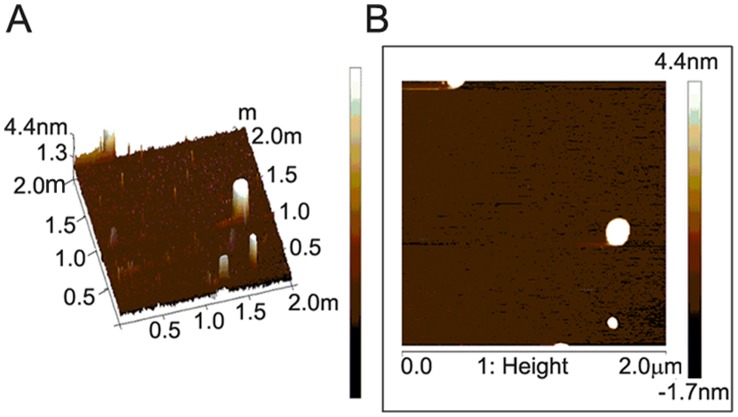
Atomic Force Microscopy micrographs of H_2_bdtc loaded in SLNs (A) 2 µm×2 µm three-dimensional image. (B) 2 µm×2 µm planar image.

The H_2_bdtc showed a lipophilic character (Log P (o/w)  = 2.69±0.03) and were efficiently encapsulated in this manner in SLNs. On the basis of Photon Correlation Spectroscopy (PCS), H_2_bdtc-SLNs had diameter of 127.4±10.2 nm and dispersity lower than 0.3; the zeta potential revealed a negative surface charge (−56.1±4.4 mV) (Table 1). The entrapment efficiency was 98.16±1.12, showing that the drug dispersed well within the lipid matrix. Atomic force microscopy images revealed that H_2_bdtc-SLNs particles were spherical, with an average diameter of approximately 180 nm (Figure 2), agreeing with the PCS results.

**Table 1:** Particle size, zeta potential, dispersity index and entrapment efficiency of SLNs (mean ± SD, n = 3).

Formulation				
Compounds	Mean Particle (nm)	D-Stroke	Zeta Potential (mV)	Entrapment Efficiency (%)
**SLN**	132.3±10.2	0.260±0.0098	−60.20±2.30	98.16±1.12
**H_2_bdtc-SLN**	127.4±0.130	0.229±0.130	−56.10±4.40	

*Dispersity (D-stroke). SLNs: SLNs without drug loaded and H2bdtc loaded in SLNs: H2bdtc loaded in SLNs.

### *In vitro* evaluation of the trypanocidal activity and cytotoxicity of free H_2_bdtc and H_2_bdtc- SLNs

**Figure 3:**
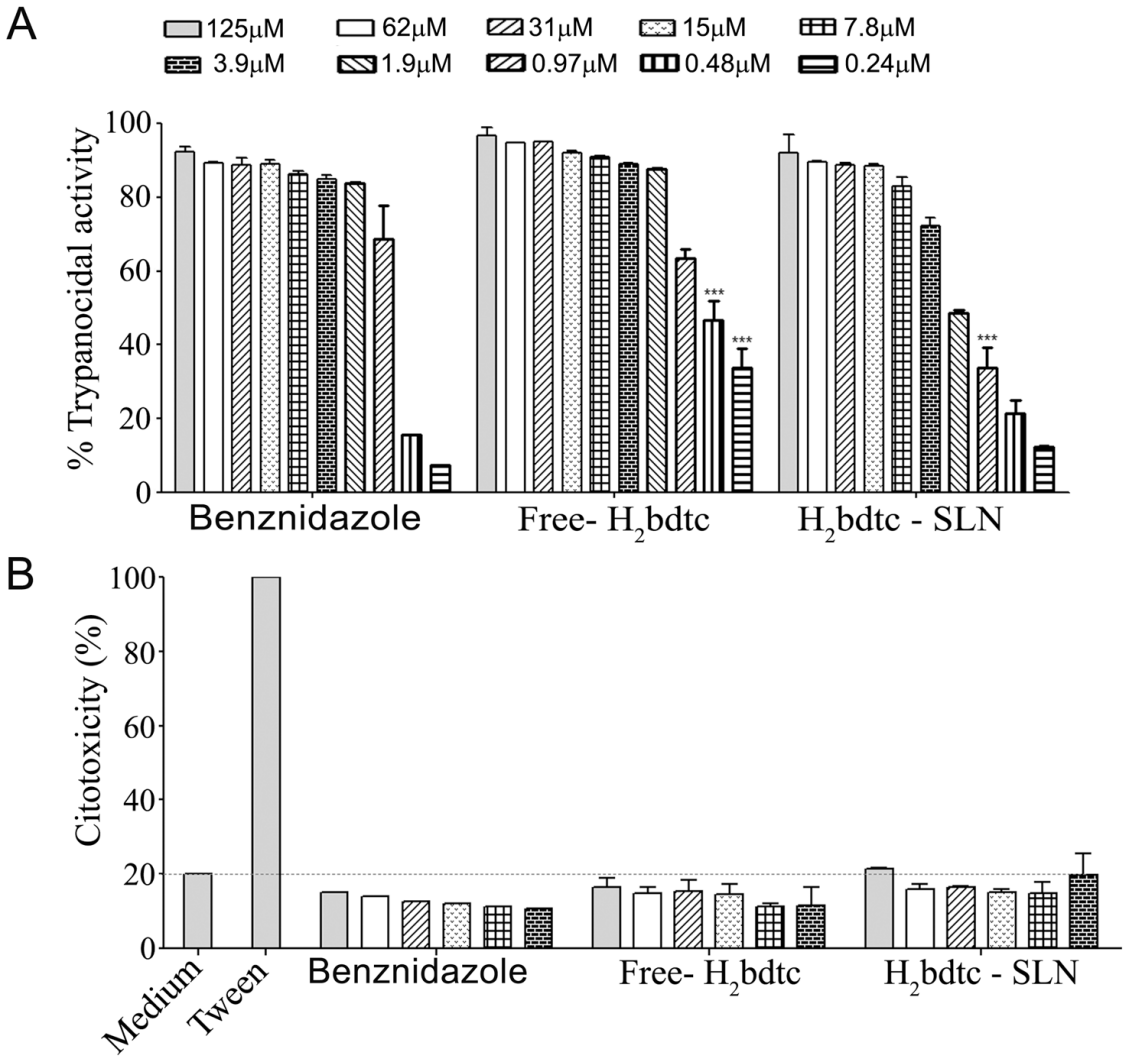
*In vitro* evaluation of the trypanocidal activity and cytotoxicity of H_2_bdtc – (concentrations range from 125 µM to 0.24 µM in serial dilutions). (A) Percentage of trypanocidal activity of free H_2_bdtc and H_2_bdtc loaded in SLNs against the Y strain of T. cruzi analyzed by quantifying viable parasites via microscopy after 24 h post-treatment. (B) Percentage of cytotoxicity of free H_2_bdtc and H_2_bdtc loaded in SLNs in spleen cells derived from mice after 24 h via propidium iodide treatment and FACS analysis. The mean + SEM is shown and is representative of three independent experiments (n = 2). Statistically significant differences compared with the control (BZN). Anova, Bonferroni post - test: ***p<0.001

We assessed the *in vitro* trypanocidal activity of free H_2_bdtc, H_2_bdtc-SLNs and BZN after 24 h of incubation with *T. cruzi* trypomastigotes forms. Free H_2_bdtc presents IC_50try_ (inhibitory concentrations against bloodstream trypomastigote) as 0.50±0.12, H_2_bdtc-SLNs as 1.83±0.18 and BZN 0.50±0.39 µM (Figure 3A). We also measured the cytotoxicity of free H_2_bdtc and H_2_bdtc-SLNs in spleen cells of Swiss mice; none of the tested drugs was significantly cytotoxic (Figure 3B). Hence, both free H_2_bdtc and H_2_bdtc-SLNs displayed similar *in vitro* trypanocidal activity to BZN; this activity was not associated with general cytotoxicity but rather with specific activity against the parasite.

### *In vivo* activity of free H_2_bdtc and H_2_bdtc-SLNs

**Figure 4:**
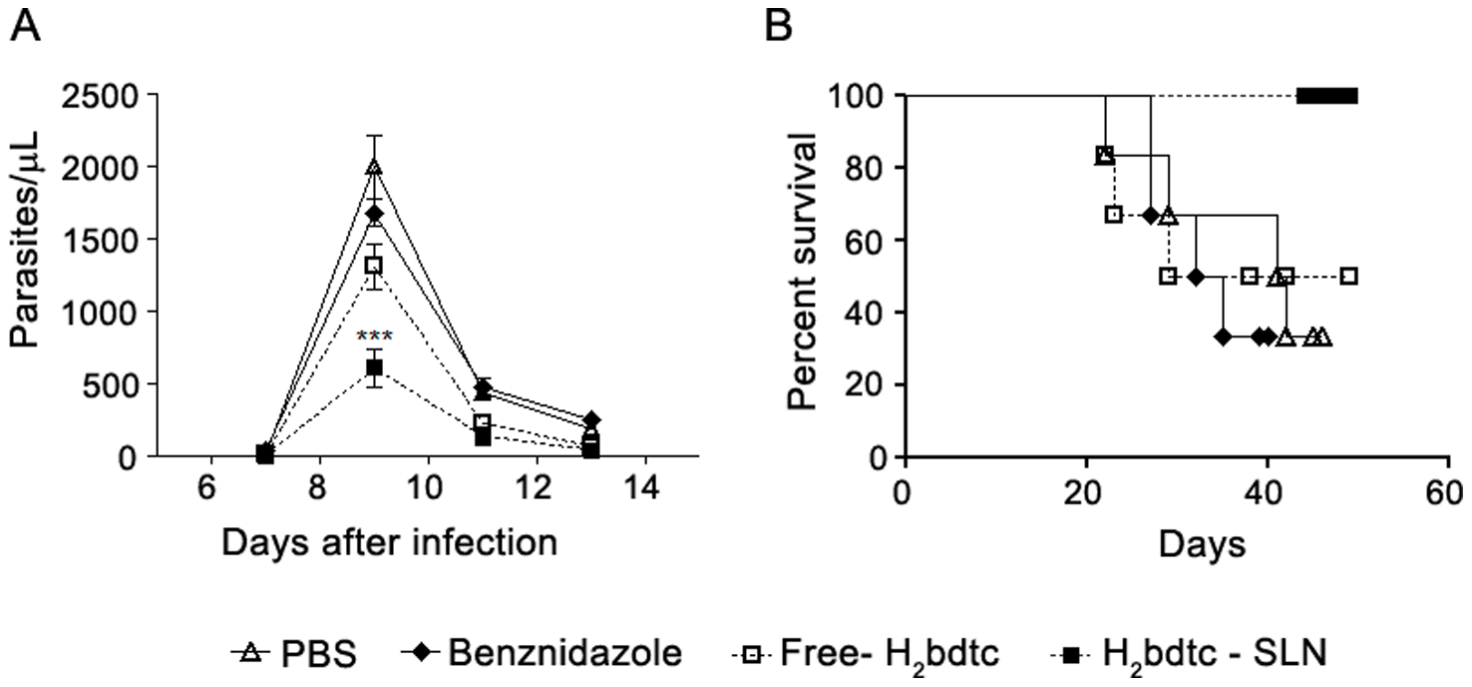
Parasitaemia and survival rates of mice infected with T. cruzi and treated with free H2bdtc, H2bdtc encapsulated in SLNs and BZN. (A) Parasitaemia was monitored on days 7, 9, 11 and 13 after infection. (B) Survival was monitored daily for 60 consecutive days. The mean + SEM is shown and is representative of three independent experiments (n = 7). Statistically significant differences compared with the control (BZN). T student test: ***p<0.001

We performed *in vivo* experiments to investigate the controlled release behavior of H_2_bdtc from H_2_bdtc-SLNs; we also compared the activities of free H_2_bdtc and H_2_bdtc-SLNs against *T. cruzi*. We decided to use an H_2_bdtc-SLNs concentration of 4 µmol kg^−1^ day^−1^. During the *in vivo* treatments on the basis of preliminary *in vivo* results obtained for the Y strain of *T. cruzi*, which revealed that H_2_bdtc had low level of parasitemia (Supporting Information: Figure S1). In all the infected groups, parasitemia peaked at day 9 p.i., with gradual parasite elimination from the bloodstream after day 11 p.i. It is worth noting that we employed BZN concentrations 100 times lower than that used for Chagas patients. H_2_bdtc-SLNs eliminated 70% of the circulating parasites at the peak of infection, whereas free H_2_bdtc and the positive control BZN eliminated 48 and 15% of the parasites, respectively, as compared with the control group treated with PBS (Figure 4A). In agreement with the data revealing reduced parasitemia, mice treated with H_2_bdtc-SLNs presented 100% survival rate (Figure 4B), similar to the result achieved with BZN administered at a clinical dose of 400 µmol kg^−1^ day^−1^ (100 times more concentrated than the concentration used herein). Compared with the control group (PBS), groups treated with free H_2_bdtc and BZN exhibited a survival rate of 57%.

### Cardiac and liver lesions

**Figure 5:**
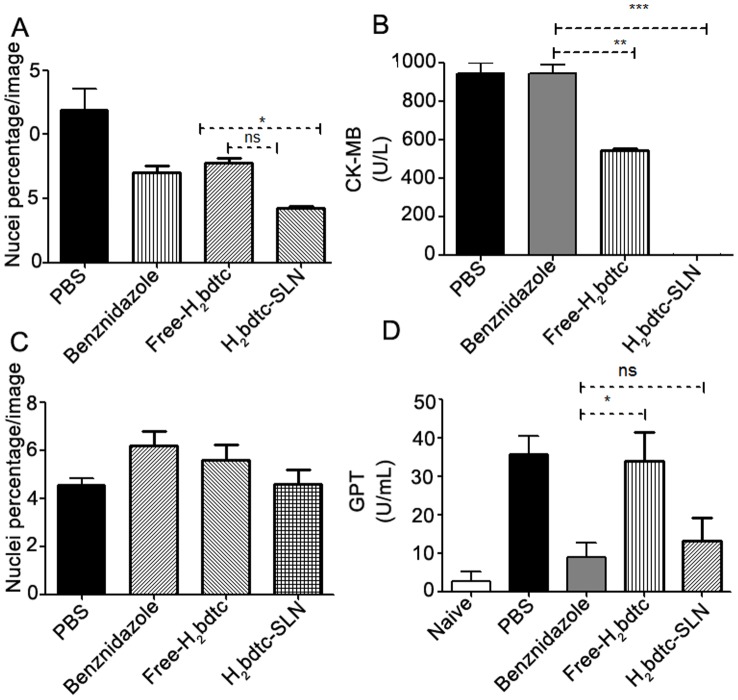
Cardiac and liver lesions of T. cruzi- infected animals after treatment with H2bdtc encapsulated in solid lipid nanoparticles. (A) Quantification of cellular nuclei per 50 µm2 of heart tissues derived from non-treated and treated animals. (B) Quantification of CK-MB in the serum of infected and treated mice. (C) Quantification of cellular nuclei per 50 µm^2^ of liver tissues derived from non-treated and treated animals. (D) Quantification of glutamic-pyruvic transaminase (GPT) levels. The mean + statistically significant differences compared with the control are denoted by: *p<0.05, **p<0.01 e ***p<0.001, T student test.

Encouraged by the *in vivo* results, we evaluated how free H_2_bdtc, H_2_bdtc-SLNs, and BZN affected the cardiac and hepatic tissues of the surviving animals. Infected mice treated with H_2_bdtc-SLNs presented reduced cardiac inflammation (Figure 5A) and heart lesions were absent (Figure 5B), as established by the absence of CK-MB, the enzyme released into plasma during cardiac lesion. Treatment with free H_2_bdtc diminished cardiac damage by 50% as compared with therapies with BZN or PBS. Concerning the ability of the tested compounds to reduce the liver damage caused by the parasite, H_2_bdtc-SLNs decreased inflammatory infiltration in the liver and hepatic toxicity more effectively, as assessed by measuring the glutamic-pyruvic transaminase (GTP) levels in the serum (Figure 5C, 5D). Considering all these results, it is possible to infer that treatment with H_2_bdtc per se reduced exacerbation of the inflammatory response on *T. cruzi* target organs and, consequently, tissue damage. Loading of H_2_bdtc into nanoparticles afforded even better results, producing no lesion in the heart tissue.

**Figure 6:**
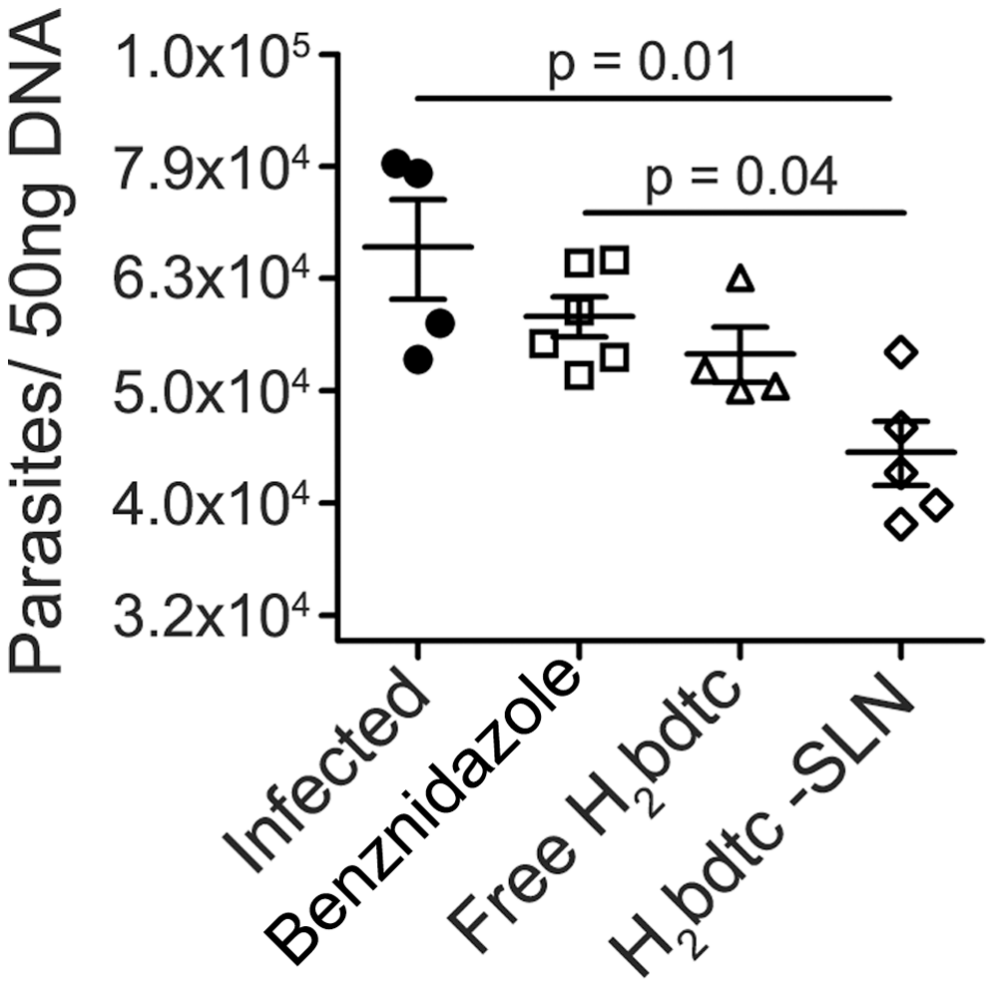
Quantification of the parasites load in cardiac tissues via real-time PCR. The presence of T. cruzi in infected heart tissue mice were analyzed by PCR 22days after infection. Statistically significant differences were showed in the figure.

Because it is well established that parasites play an important role in cardiac damage during *T. cruzi* infection [42], [43], we quantified *T. cruzi* DNA derived from the heart tissues of mice treated with the tested compounds via real-time PCR. Treatment with H_2_bdtc-SLNs reduced the parasite burden significantly more effectively as compared with the other tested drugs (Figure 6), indicating that killing the parasites is most likely the mechanism through which H_2_bdtc-SLNs acts to diminish tissue lesions and enhance mice survival.

## Discussion

Chagas disease has often been pointed out as being a major neglected disease; the drugs that are currently available to treat this disease are little effective [5], [12]. Efforts have been made to provide the affected populations with new compounds to treat the disease. In the past few years, researchers have tested many substances against *T. cruzi*. In particular, H_2_bdtc, which belongs to the class of S-dithiocarbazates, is efficient against the parasite [21]. A 24-h UV-vis study into the stability of H_2_bdtc in aqueous solution did not evidence any changes in the spectrum of this compound. Nevertheless, this drug is poorly soluble in water (1.50×10^−6^ M), which has limited its use to treat Chagas disease. Because H_2_bdtc is lipophilic (Log *P*_(o/w)_  = 2,69±0,03) and SLNs constitute effective oral drug delivery systems, we loaded H_2_bdtc into this type of lipid.

We prepared the SLNs by the microemulsion method [31], [44], to avoid the use of organic solvents. The resulting SLNs had diameter of approximately 120 nm, dispersity lower than 0.3, and spherical shape, which made these lipids suitable for oral administration [45], [46], [47], [48]. The zeta potential measurement allowed us to predict the stability of the colloidal dispersion. Charged particles have high zeta potential–negative or positive–and usually do not aggregate [49]. The zeta potential results revealed that the SNPs prepared here had a negative surface charge (−56.1±4.4 mV), indicating that the system was physically stable. Loaded and unloaded SNPs had similar zeta potentials, attesting that the tested drug was completely and uniformly dispersed inside the lipid matrix [50].

Encapsulation did not change the *in vitro* trypanocidal activity of H_2_bdtc, which was higher than the activity of BZN at the same concentration used here. The IC_50_ values obtained for H_2_bdtc against the trypomastigote form of the Y strain of *T. cruzi* were comparable with or superior to those of previously reported active compounds [51], [52]. Other papers have also described the use of colloidal drug carriers such as liposomes and nanoparticles to treat Chagas diseases [53], [54]. Treatment of *T. cruzi* infection with a BZN-loaded liposome increased BZN levels in the liver and blood. Intravenous administration of free BZN and BZN-encapsulated liposome at 0.2 mg of BZN per kilogram of body weight revealed three-fold higher BZN accumulation in the liver in the second case. Nevertheless, encapsulation failed to improve the *in vivo* BZN efficacy [55].

Liposome instability prevents their use as drug delivery systems [56]. Fortunately, we verified that free H_2_bdtc and H_2_bdtc-SLNs were not toxic to the spleen cells of Swiss mice, which encouraged us to directly test the effect of H_2_bdtc formulations *in vivo* using a murine model of acute Chagas disease.

Treatment started with a relatively low oral dose of free H_2_bdtc and H_2_bdtc-SLNs (4 µmol kg^−1^ day^−1^) as compared with currently employed doses of the commercially available BZN and compounds tested in the literature [57], [58]. H_2_bdtc-SLNs, Free H_2_bdtc and BZN reduced the presence of parasites in the blood of infected mice in 70, 48 and 15% respectively. H2bdtc-SLNs maintained 100% survival rate of infected mice, whereas 43% of the mice treated with free H_2_bdtc or BZN at the same concentration succumbed to the disease. It is noteworthy that free SLN and PBS elicited similar levels of parasitemia (Supporting Information: Figure S2). Therefore, the use of SLNs as a drug delivery system increased the oral bioavailability of the target drug, as previously described [59], [60], [61], [62], [63]. H_2_bdtc loading into SLNs overcame the problems inherent to the poor water solubility of the compound and may be could make it more accessible to the parasite (however, detailed pharmacokinetic data will be presented in a separate forthcoming paper). Additionally, some authors have proposed that drugs loaded into SLNs measuring 20–500 nm are absorbed by lymphatic transport, which reduces the first-pass metabolism [62], [63].

Analysis of histological sections of liver and heart tissues (Supporting Information: Figure: Figure S3 and Figure S4) revealed that the inflammatory infiltrate decreased in all the treated groups as compared with the control. The reduction was more pronounced in mice treated with H_2_bdtc-SLNs, possibly because parasitemia was lower in this case. This corroborated with findings from previous studies [64], [65] and confirmed that the parasite elicited intense inflammation especially in the cardiac tissues. The fact that the heart tissues of mice treated with H_2_bdtc-SLNs were perfectly preserved agreed with the notion that the presence of inflammatory infiltrates is associated with cardiac tissue damage [66], [67] and also with parasitic load [42], [43]. Indeed, mice treated with H_2_bdtc-SLNs exhibited significantly lower parasite burden as compared with the other groups. Hence, the reduced parasitism elicited by H_2_bdtc-SLNs helps to preserve the heart tissues of mice infected with *T. cruzi*, allowing us to conclude that H_2_bdtc is a potent trypanocidal agent.

Investigation into how H_2_bdtc interacts with possible targets represents a theme for future studies. For the time being, we must bear in mind that triazoles and thiosemicarbazones are well known for inhibiting cruzain, a protein belonging to the family of cysteine proteases and which is the most abundant protein in *T. cruzi*. Cruzain is essential for parasite development and survival within host cells [68]. H_2_bdtc bears pyrazole and dithiocarbazate parts, which are similar to triazoles and thiosemicarbazones, respectively, and could account for its trypanocidal action.

A mechanism of action similar to that of BZN probably does not occur. The BZN mode of action involves intracellular reduction of the nitro group, to produce highly reactive free radicals and/or electrophilic metabolites that could affect other systems, especially host systems, contributing to the cytotoxic effects observed in BZN-treated patients [69].

It is worth noting that cysteine proteases are very important for parasites; however, the lack of redundancy with respect to their mammalian hosts makes these proteases interesting targets for the development of new therapeutic agents [70]. Altogether, our findings show that H_2_bdtc-SLNs are a possible drug candidate to treat Chagas disease: it is more efficient against *T. cruzi* than the drugs used in current therapies.

## Supporting Information

Figure S1 *In vivo* evaluations of the trypanocidal activity free-H_2_bdtc and H_2_bdtc-SLNs – (concentrations 4 µM, 40 µM and 80 µM) (^_____^) Parasitaemia rate of mice infected with T. cruzi and treated with free-H_2_bdtc. (----) Parasitaemia rate of mice infected with T. cruzi and treated with H_2_bdtc encapsulated in solid lipid nanoparticles. Parasitaemia was monitored on days 7, 9, 11 and 13 after infection. The mean + SEM is shown and is representative of three independent experiments (n = 7). Statistically significant differences compared with the free-H_2_bdtc. T student test: **p<0.01 and ***p<0.001.
(TIF)

Figure S2 Parasitaemia of mice infected with T. cruzi and treated with free SLNs and PBS. Parasitaemia was monitored on days 7, 9, 11 and 13 after infection.
(TIF)

Figure S3 Cardiac lesions of T. cruzi-infected animals after treatment with H2bdtc encapsulated in SLNs. The sections represent of heart tissues inflammatory process composed of various cell types (21 days after infection).
(TIF)

Figure S4 Liver lesions of T. cruzi-infected animals after treatment with H_2_bdtc encapsulated in SLNs. The sections represent of liver tissues inflammatory process composed of various cell types (21 days after infection).
(TIF)
