# Correction: *In Vitro* and *In Vivo* Trypanocidal Activity of H2bdtc-Loaded Solid Lipid Nanoparticles

**DOI:** 10.1371/journal.pntd.0003524

**Published:** 2015-02-23

**Authors:** The PLOS Neglected Tropical Diseases Staff

There is an error in reference 49. The correct reference is: Doostmohammadi A, Monshi A, Salehi R, Fathi MH, Karbasi S, Pieles U, Daniels AU (2012) Preparation, chemistry and physical properties of bone-derived hydroxyapatite particles having a negative zeta potential. Mater Chem Phys 132: 446–452 doi:10.1016/j.matchemphys.2011.11.051.
