# Supplementary figures and images for: In Vitro and In Vivo Trypanocidal Activity of H2bdtc-Loaded Solid Lipid Nanoparticles
Source: PLoS Negl Trop Dis. 2014 May 8;8(5):e2847. doi: 10.1371/journal.pntd.0002847 (PMC4014426; doi:10.1371/journal.pntd.0002847)

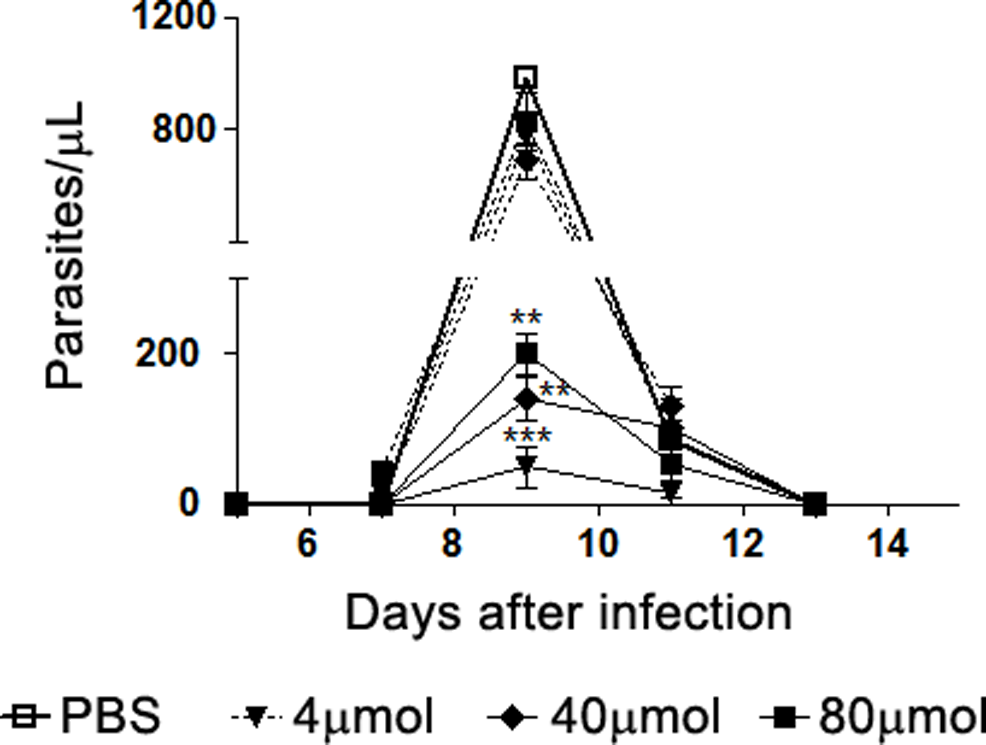

Supplement: Figure S1 — In vivo evaluations of the trypanocidal activity free-H2bdtc and H2bdtc-SLNs – (concentrations 4 µM, 40 µM and 80 µM) (_____) Parasitaemia rate of mice infected with T. cruzi and treated with free-H2bdtc. (----) Parasitaemia rate of mice infected with T. cruzi and treated with H2bdtc encapsulated in solid lipid nanoparticles. Parasitaemia was monitored on days 7, 9, 11 and 13 after infection. The mean + SEM is shown and is representative of three independent experiments (n = 7). Statistically significant differences compared with the free-H2bdtc. T student test: **p<0.01 and ***p<0.001. (TIF) [file pntd.0002847.s001.tif]

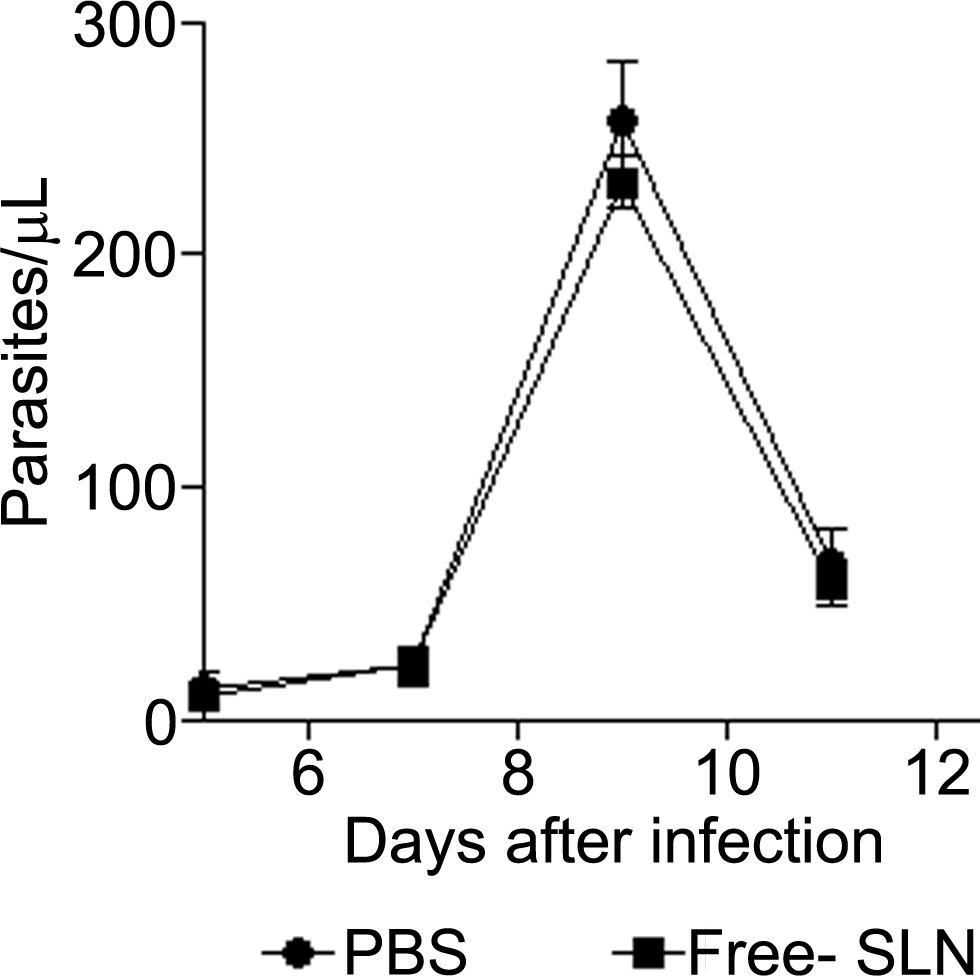

Supplement: Figure S2 — Parasitaemia of mice infected with T. cruzi and treated with free SLNs and PBS. Parasitaemia was monitored on days 7, 9, 11 and 13 after infection. (TIF) [file pntd.0002847.s002.tif]

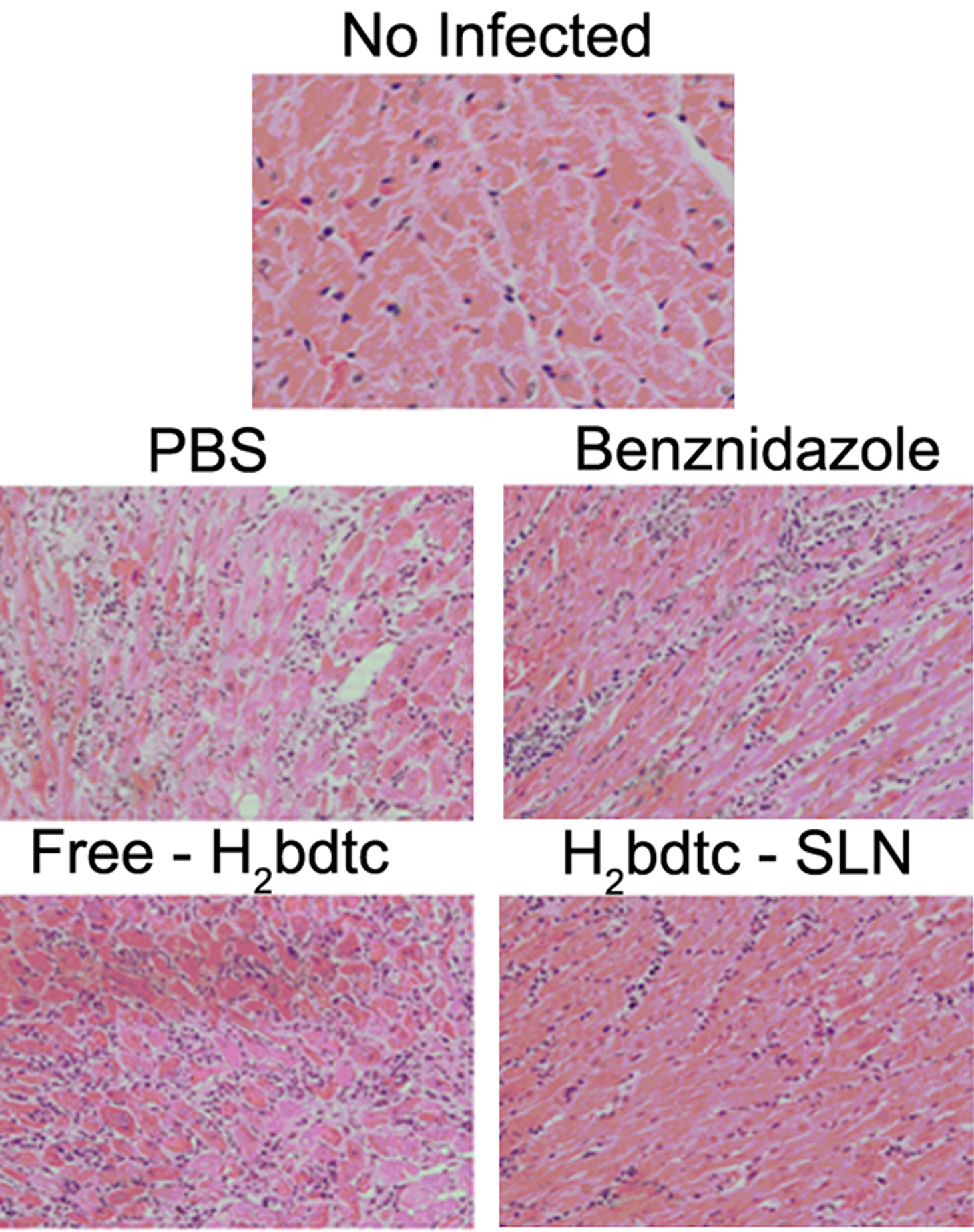

Supplement: Figure S3 — Cardiac lesions of T. cruzi-infected animals after treatment with H2bdtc encapsulated in SLNs. The sections represent of heart tissues inflammatory process composed of various cell types (21 days after infection). (TIF) [file pntd.0002847.s003.tif]

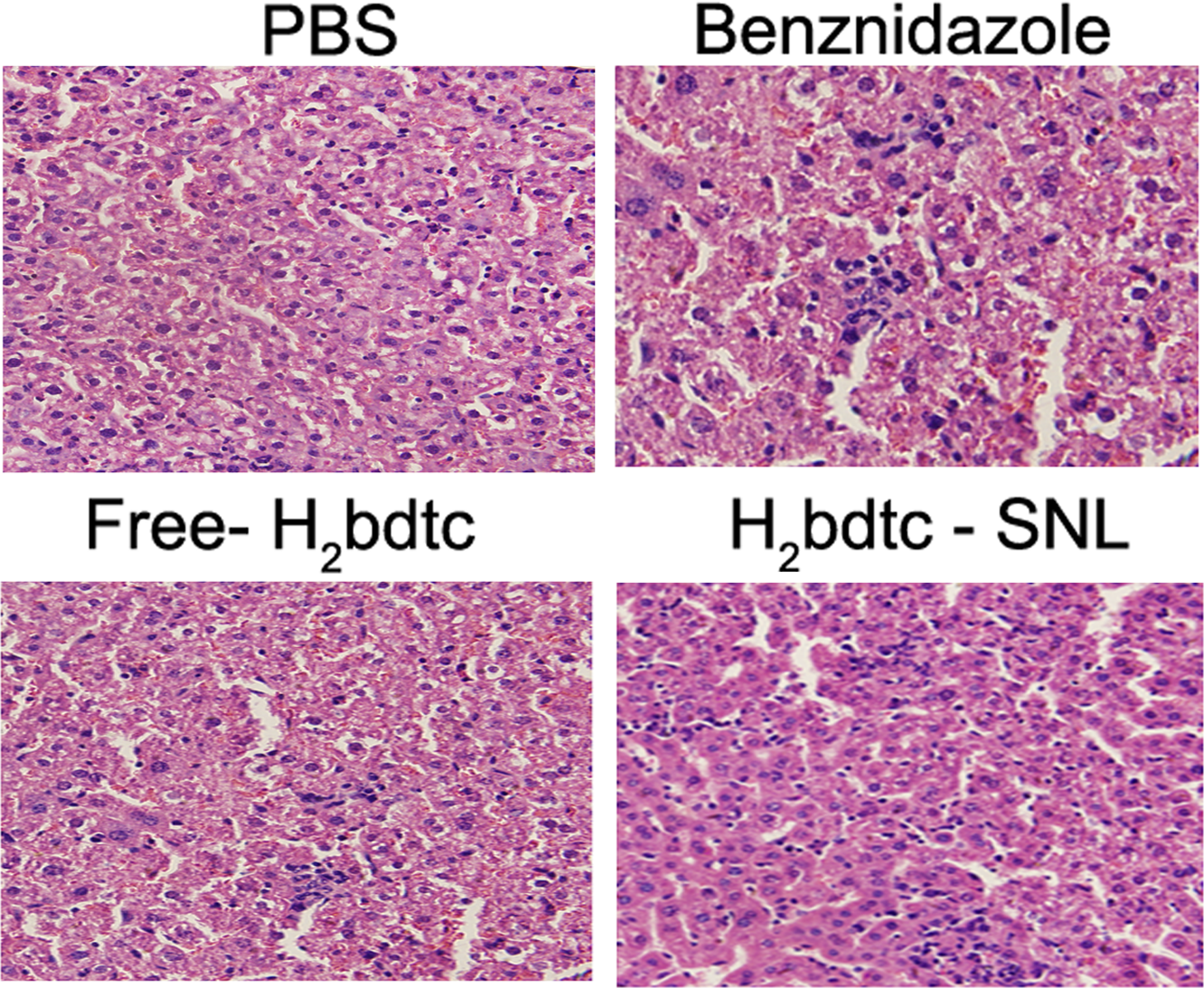

Supplement: Figure S4 — Liver lesions of T. cruzi-infected animals after treatment with H2bdtc encapsulated in SLNs. The sections represent of liver tissues inflammatory process composed of various cell types (21 days after infection). (TIF) [file pntd.0002847.s004.tif]
